# MCCC2 is a novel mediator between mitochondria and telomere and functions as an oncogene in colorectal cancer

**DOI:** 10.1186/s11658-023-00487-0

**Published:** 2023-10-12

**Authors:** Wanjun Liu, Si Chen, Wenqing Xie, Qian Wang, Qianxin Luo, Minghan Huang, Minyi Gu, Ping Lan, Daici Chen

**Affiliations:** 1 Guangdong Provincial Key Laboratory of Colorectal and Pelvic Floor Diseases, The Sixth Affiliated Hospital, Sun Yat-sen University, 26 Yuancun Er Heng Road, Guangzhou, 510655 Guangdong China; 2 Biomedical Innovation Center, The Sixth Affiliated Hospital, Sun Yat-sen University, Guangzhou, China; 3 Department of Intensive Care Unit, The Sixth Affiliated Hospital, Sun Yat-sen University, 26 Yuancun Er Heng Road, Guangzhou, 510655 China; 4 Scientific Journal Center, The Sixth Affiliated Hospital, Sun Yat-sen University, 26 Yuancun Er Heng Road, Guangzhou, 510655 China; 5 Department of General Surgery, The Sixth Affiliated Hospital, Sun Yat-sen University, 26 Yuancun Er Heng Road, Guangzhou, 510655 China

**Keywords:** MCCC2, Mitochondria, Telomere, Colorectal cancer

## Abstract

**Background:**

The mitochondrial gene MCCC2, a subunit of the heterodimer of 3-methylcrotonyl-CoA carboxylase, plays a pivotal role in catabolism of leucine and isovaleric acid. The molecular mechanisms and prognostic value still need to be explored in the context of specific cancers, including colorectal cancer (CRC).

**Methods:**

In vitro and in vivo cell-based assays were performed to explore the role of MCCC2 in CRC cell proliferation, invasion, and migration. Mitochondrial morphology, membrane potential, intracellular reactive oxygen species (ROS), telomerase activity, and telomere length were examined and analyzed accordingly. Protein complex formation was detected by co-immunoprecipitation (CO-IP). Mitochondrial morphology was observed by transmission electron microscopy (TEM). The Cancer Genome Atlas (TCGA) CRC cohort analysis, qRT-PCR, and immunohistochemistry (IHC) were used to examine the MCCC2 expression level. The association between MCCC2 expression and various clinical characteristics was analyzed by chi-square tests. CRC patients’ overall survival (OS) was analyzed by Kaplan–Meier analysis.

**Results:**

Ectopic overexpression of MCCC2 promoted cell proliferation, invasion, and migration, while MCCC2 knockdown (KD) or knockout (KO) inhibited cell proliferation, invasion, and migration. MCCC2 KD or KO resulted in reduced mitochondria numbers, but did not affect the gross ATP production in the cells. Mitochondrial fusion markers MFN1, MFN2, and OPA1 were all upregulated in MCCC2 KD or KO cells, which is in line with a phenomenon of more prominent mitochondrial fusion. Interestingly, telomere lengths of MCCC2 KD or KO cells were reduced more than control cells. Furthermore, we found that MCCC2 could specifically form a complex with telomere binding protein TRF2, and MCCC2 KD or KO did not affect the expression or activity of telomerase reverse transcriptase (TERT). Finally, MCCC2 expression was heightened in CRC, and patients with higher MCCC2 expression had favorable prognosis.

**Conclusions:**

Together, we identified MCCC2 as a novel mediator between mitochondria and telomeres, and provided an additional biomarker for CRC stratification.

**Supplementary Information:**

The online version contains supplementary material available at 10.1186/s11658-023-00487-0.

## Background

Colorectal cancer (CRC) is a malignant tumor of the digestive tract, that is, of the colon and rectum. CRC is the third most commonly diagnosed cancer and second most common cause of cancer-related death worldwide [1, 2]. It has been reported that approximately 50% of patients with CRC eventually develop metastases, and uncontrollable metastasis is the main cause of death [3–5]. Currently, the standard treatment for CRC is surgery. In addition to chemotherapy, including targeted therapy, radiotherapy is commonly used in combination with surgery to treat patients with CRC [2, 6]. The 5-year overall survival (OS) rate for stage I and stage II could exceed 90%, but the 5-year OS rate drops to less than 25% in patients with advanced stages (metastasized) [7]. Therefore, a toolkit with various biomarkers is valuable for early diagnosis and prognostic prediction for CRC patients.

Cancer cells are characterized by various hallmarks during which both telomeres and mitochondria play vital roles. It is well known that cancer cells can shift metabolism and redox homeostasis as needed, which are rigorously related to mitochondria dynamics [8]. The important mechanical changes in mitochondrial architecture encompass both fusion and fission, which are mediated by specific proteins located in the outer and inner membranes, such as mitofusin (MFN) 1 and 2, optic atrophy 1 (OPA1), and dynamin-related protein 1 (DRP1) [9, 10]. A previous study showed that mitochondrial fission could be beneficial to cellular migration and therefore tumor progression [11]. Meanwhile, inhibiting mitochondrial fusion with MFN1, MFN2, or OPA1 deficiencies results in severe cellular defects, including widespread heterogeneity of the mitochondrial membrane potential, decreased cellular respiration, and poor cell growth [12]. Therefore, mitochondrial fusion–fission dynamics can affect cell physiology and are strictly controlled.

The human chromosome end is capped by telomeres to prevent degradation by nucleolytic attack and to distinguish chromosome termini from double-strand break (DSB) [13]. Telomeric tandem repeat of hexameric (TTAGGG) DNA is assembled into a T-loop structure with the help of the shelterin complex, a hetero-hexamer composed of TRF1, TRF2, TIN2, POT1, TPP1, and RAP1. The length of the telomere progressively shortens with each cell division, and telomerase reactivation is a hallmark of cancer cells, which enables replicative immortality [14]. Telomerase reverse transcriptase (TERT) and telomeric RNA component (TERC) are two main components of the telomerase ribonucleoprotein complex. While TERT protein is mainly localized in the nucleus, 10–20% of total TERT is localized in the mitochondria. The non-telomeric functions of TERT include decreased ROS, DNA damage, and apoptosis [15, 16]. A recent study showed that TERC was imported into mitochondria, processed to a shorter form of TERC-53, and then exported back to the cytosol [17]. However, damage to mitochondria, for example, by inducing mitochondrial ROS, could also cause rapid telomere dysfunction [18]. In addition, the generation of ROS through aberrant mitochondrial activity could induce single-strand breaks at telomeres, and both contribute to dysfunctional telomeres [19]. Moreover, telomere dysfunction leads to p53-dependent repression of PGCα/β, resulting in decreased ATP synthesis, elevated ROS levels, and decreased metabolic capacity, including impaired gluconeogenesis [20]. These results suggest that cross-talk exists between telomeres and the mitochondria. Therefore, identifying more mediators between mitochondria and telomeres is pivotal for understanding the underlying molecular basics, especially in the context of tumorigenesis and progression.

Mitochondrial methylcrotonoyl-CoA carboxylase is a heterodimeric enzyme (coding by MCCC1 and MCCC2 gene, respectively) that catalyzes the conversion of 3-methylcrotonyl-CoA to 3-methylglutaconyl-CoA, a critical step for leucine and isovaleric acid catabolism. Genetic mutations in either MCCC1 or MCCC2 in newborn babies would cause 3-methylcrotonyl-CoA carboxylase deficiency during leucine catabolism, resulting in an organic aciduria situation called 3-methylcrotonylglycinuria [21–23]. Recently, MCCC2 has been implicated as an oncogenic factor in various cancers. MCCC2 is upregulated in breast and colorectal cancer [24, 25]. Related to its enzyme activity, by supporting leucine oncogenic function, MCCC2 promoted HCC development [26]. Nonetheless, the molecular basis in which MCCC2 functions in cancer progression is still unclear. In the current study, we found that MCCC2 promotes proliferation, invasion, and migration of CRC cell lines. Furthermore, we found that MCCC2 deficiency not only leads to mitochondrial fusion, which may contribute to maintaining the mitochondrial membrane potential and compensating for energy production, but also causes telomere shortening, suggesting that MCCC2 is a novel mediator between mitochondria and telomeres. We found that MCCC2 was upregulated in CRC. Interestingly, higher MCCC2 expression is associated with longer OS in our cohort study, which is consistent with the results of TCGA-CRC cohort analysis.

## Methods

### Patients’ clinical information and tissue sample

CRC patients (*n* = 41) with primary CRC tissues and matched adjacent normal tissues were obtained from the biological sample bank of the Sixth Affiliated Hospital, Sun Yat-sen University (SYSU).

We also acquired primary CRC and paired adjacent normal tissues from 233 patients from the Six Affiliated Hospital of SYSU and conducted tissue microarrays. Before surgical resection of the tumor, none of the patients received chemotherapy, radiotherapy, or other related treatments. This study was approved by the Institutional Ethics Committee of the Six Affiliated Hospital, Sun Yat-sen University, and performed in accordance with the Declaration of Helsinki.

### Immunohistochemistry (IHC) assay

Tissue microarray and related clinicopathological information were collected from the Sixth Affiliated Hospital of SYSU. IHC for MCCC2 was performed on the CRC tissue microarray slides. The slides were first incubated at 60 ℃ for 4–6 h, next deparaffinized with xylene, rehydrated with decreasing ethanol concentrations, and then heated in citrate buffer for 25 min. When dropped to room temperature, an IHC kit (cat. no. SP9000; ZSGB-Bio) was used to block endogenous peroxidase activity. Slides were blocked with 5% goat serum for 1 h and incubated with anti-MCCC2 antibody (1:500, cat. no. HPA038300, RRID:AB_2675941, Sigma) overnight at 4 ℃. The next day, after three washes with PBST, the slides were incubated with secondary antibody at room temperature for 1–2 h and then stained with a DAB kit (cat. no. ZLT-9017, ZSGB-Bio). After the experiments, slides were observed under a microscope. The IHC scores were independently assessed by two pathologists. In accordance with the ROC curve analysis, an IHC score of 10.3 was selected as the cutoff value that divided the cohort in to two groups: MCCC2 low expression (*n* = 98) and MCCC2 high expression (*n* = 112).

### Cell lines and plasmids

All the cell lines used in this study were obtained from the American Type Culture Collection (ATCC Manassas, VA, USA). The cells were maintained in DMEM (Gibco) supplemented with 10% Fetal Bovine Serum (Gibco) and typically cultured at 37 ℃ in a humidified incubator with 5% CO_2_. HCT116 cells were transfected with lentiviruses containing the relevant shRNA or FLAG-MCCC2 plasmid. Puromycin dihydrochloride (2 μg/mL) was used to treat and screen cells. Wild-type MCCC2 (NM_022132.4, amino acids 1–564) gene, mutant MCCC2 (amino acids ∆2–22, 543aa; amino acids ∆343–372, 534aa) were cloned into the pEZ-Lv242 vector (1 × FLAG-tagged). The shRNA sequences targeting MCCC2 were cloned into the psi-LVRU6GP vector (EGFP). To generate MCCC2 KO HCT116 cells, three sgRNAs targeting exon 1 of MCCC2 were cloned into the pCRISPR-CG041 vector. Empty vectors were purchased from GeneCopoeia (FulenGen Co., Ltd., Guangzhou, China).

shMCCC2 sequences: 5′-GCAGGTTACCAGTTATATGAC-3′.

siMCCC2-1: 5′-GGGCCCAAGAAATTGCCAT-3′.

siMCCC2-2: 5′-GGATCTTGGAGGTGCTGAT-3′.

### Generation of MCCC2 Knockout cells

HCT116 cells were transfected with the three pCRISPR-CG041-a/b/c vectors at a ratio 1:1:1 using Lipofectamine^®^ 3000 reagent. The cells were cultured with neothramycin (800 μg/mL) for 5 days. The positive cells were planted into 96-well plates to isolate single-cell clones, whose DNA was extracted and subjected to T7 endonuclease survey assay and subsequent Sanger sequencing.

MCCC2 sgRNA-a: 5′- GCCGGGCCGCGCGCCTATCA-3′.

MCCC2 sgRNA-b: 5′-CCGCCATGTGGGCCGTCCTG-3′.

MCCC2 sgRNA-c: 5′-CTTGGGCTCTGCCCTCTACC-3′.

### Western blotting

Cellular proteins were extracted using routine cell lysis solution and boiled in 1 × SDS loading buffer for 10 min. Proteins were separated by SDS-PAGE (8% or 10% separation gel) and transferred to PVDF membranes. The membranes were probed with antibodies against MCCC2 (1:1000 dilution, cat. no. HPA038300, RRID:AB_2675941, Sigma), MFN1 (1:1000 dilution, cat. no. AP16037c, Abcepta), MFN2 (1:1000 dilution, cat. no. AP8840c, Abcepta), OPA1 (1:1000 dilution, cat. no. AP20727c, Abcepta), FLAG (1:1000 dilution, cat. no. 390002, ZEN-BIOSCIENCE), and TERF2 (1:1000 dilution, cat. no. 22020-1-AP, Proteintech). GAPDH (1:2000 dilution, cat. no. 390035, ZEN-BIOSCIENCE) and α-tubulin (1:20,000 dilution, cat. no. 66031–1-Ig, Proteintech) antibodies were used as loading controls.

### Cell proliferation assay

The cells were counted and seeded at a density of 5000 cells/well in 96-well plates. A real-time cell analyzer (RTCA, xCELLigence system, ACEA Biosciences, USA) and IncuCyte (Zoom, Essen Bioscience, USA) were used to monitor the cell growth. The live cells were recorded automatically every 120 min, and fresh culture medium was exchanged every 3 days. The experiments were conducted in triplicates.

### Colony formation assay

Five hundred cells were counted and seeded in six-well plates for 2 weeks in a humidified incubator with 5% CO_2_, and the medium was exchanged every 3 days. The cells were washed with PBS and fixed with 4% paraformaldehyde for 30 min. The colonies were stained with 0.1% crystal violet for 15 min and washed with PBS. The experiments were conducted in triplicates.

### Wound-healing assay

Cells were seeded into 24-well plates, which were placed in a 4-well silicone insert (Ibidi, Martinsried, Germany) per well, and incubated until 100% confluence in each well. After removing the silicone insert, the cells were cultured in an FBS-free culture medium. Live cell images were collected in an IncuCyte Essens Bioscience incubator every 120 min.

### Transwell invasion assays

Cell invasion assays were performed in Transwell chambers containing 8-μm Transwell filters (Corning, NY, USA) placed in 24-well plates. The upper chambers were covered with Matrigel (Corning, NY, USA) and placed in at 37 ℃ incubator for 1 h. Then, 1 × 10^5^ cells were counted, seeded into upper chamber, and incubated with FBS culture medium at 37 ℃ for 36–48 h. The upper chamber was wiped with cotton swabs, and the invading cells were attached to the lower surface of the membrane. The cells were fixed with 4% paraformaldehyde and stained with crystal violet for 5 min. Photographs were obtained using a brightfield microscope (Olympus, Japan).

### Co-immunoprecipitation (Co-IP)

Whole-cell protein lysates were prepared using a cell lysis solution (NP-40, phosphatase inhibitors, and protease inhibitors). Nuclear proteins were extracted using a nuclear and cytoplasmic extraction kit, according to the manufacturer’s instructions (cat. no. P0027, Beyotime). Fifty microliters of Protein A/G magnetic beads (cat. no. HY-K0202, MCE) were incubated with ~ 2 μg antibody or IgG at 4 ℃ for 2–3 h, then incubated with cell lysates at 4 ℃ overnight. The complexes were washed with lysis buffer for eight times before preparation for immunoblotting.

### Mitochondrial staining for quantity measurement, JC-1 assay, and intracellular ROS assay by flow cytometry

Cells were incubated with 100 nM MitoTracker Red CMXRos (cat. no. C1035, Beyotime) in HBSS (Ca^+^ and Mg^+^ free) at 37 ℃ for 30 min. The cells were subjected to a CytoFLEX flow cytometer (Beckman Coulter, USA) to determine the mitochondria-targeted red fluorescent probe for quantification at an excitation wavelength of 549 nm and analyzed using CytExpert software (Beckman Coulter, USA).

Cells were detected using an enhanced mitochondrial membrane potential assay kit with JC-1 (cat. no. C2003S, Beyotime), according to the manufacturer’s instructions. Briefly, the precipitated cells (~ 5 × 10^5^ cells) were harvested and resuspended in 500 µL of cell culture medium. The cell suspension was incubated with 500 µl JC-1 at 37 ℃ for 20 min in the dark. The cell pellet was washed with JC-1 dyeing washing buffer for four times, then resuspended in 1 mL dyeing washing buffer. The prepared cellular samples were immediately loaded onto a CytoFLEX flow cytometer (Beckman Coulter, USA) and analyzed using the CytExpert software (Beckman Coulter, USA).

Intracellular ROS was measured using an oxidation-sensitive fluorescent probe (DCFH-DA) and a Reactive Oxygen Species Assay Kit (cat. no. S0033S, Beyotime). The non-fluorescent molecule (DCFH-DA), which is deacetylated intracellularly by nonspecific esterase, is robustly oxidized to fluorescent DCF in the presence of intracellular ROS. The fluorescence intensity reflects the level of oxidative stress. The cell precipitate (~ 5 × 10^5^ cells) was harvested and resuspended in 10 µM DCFH-DA dissolved in cell-free medium at 37 ℃ for 30 min and then washed three times with PBS. The prepared cellular samples were immediately loaded onto a CytoFLEX flow cytometer (Beckman Coulter, USA) at an excitation wavelength of 488 nm and analyzed using CytExpert software (Beckman Coulter, USA).

### Measurement of cellular ATP levels

Cellular ATP levels were assayed using an ATP bioluminescence assay kit (cat. no. S0026, Beyotime) according to the manufacturer’s instructions. Briefly, cells were lysed and centrifuged at 12,000 rpm for 5 min at 4 ℃. The supernatants were mixed with the working dilution of ATP detection in a white 96-well plate. The luminance (RLU) was measured using a Thermo Scientific Varioskan Flash instrument.

### Telomeric restriction fragment (TRF) analysis

Genomic DNA was isolated using the AxyPrep Genomic DNA Miniprep Kit (cat. no. AP-MN-MS-GDNA-4, Axygen). Four micrograms of genomic DNA was digested with *Rsa*I and *Hinf*I before being resolved by electrophoresis (0.7% agarose gel, 14 h, 1.5 V/cm), The agarose gel was dried for 30 min at 50 ℃ and hybridized with radiolabeled ^32^P and telomere probe (TTAGGG)_4_. The signal intensity was quantified using a phosphor screen (Typhoon, GE). The average telomere length was analyzed and calculated using ImageJ software (RRID:SCR_003070) and ImageQuant TL.

Average TRF length was calculated from the scanned image from phosphorImager. Briefly, a grid of 150 boxes was divided over each lane, and the signal intensity for each box were calculated. The signal intensity could reflect the number of repeats in the certain molecular weight. Average TRF length could be obtained by weighted calculation [27].

### qRT-PCR, Q-TRAP

Total RNA was extracted using the RNA-Quick Purification Kit (cat. no. ES-RN001, esunbio) and quantified using Nanodrop 100 (Thermo Fisher Scientific, USA). A total of 500 ng RNA was reverse-transcribed using the ReverTra Ace^®^ qPCR RT Master Mix with a gDNA remover kit (cat. no. FSQ-301, TOYOBO). Quantitative polymerase chain reaction (qPCR) was performed using GoTaq^®^ qPCR Master Mix (Promega) on an ABI Quantistudio 7 Flex real-time PCR system. Quantification was performed using comparative CT (delta-delta CT). The primers used were:

MCCC2 FP: 5′-GCCATGGCTGATGAAAACAT-3′

MCCC2 RP: 5′-TCAGCACCTCCAAGATCCTC-3′

MFN1 FP: 5′-CAAGGTGAATGAGCGGCTTTCCAA-3′

MFN1 RP: 5′-ATGCAGGCATCTTTCCATGTGCTG-3′

MFN2 FP: 5′-TGTCTGGGACCTTTGCTCATCTGT-3′

MFN2 RP: 5′-TTCCTGAGCAGCTTTGCTTTGCTC-3′

OPA1 FP: 5′-GCATGCTAAAGGCACACCAAGTGA-3′

OPA1 RP: 5′-TTCCCGCAGGCGAGGATAGTTATT-3′

β-Actin FP: 5′-TTGTTACAGGAAGTCCCTTGCC-3′

β-Actin RP: 5′-ATGCTATCACCTCCCCTGTGTG-3′

A qPCR-based telomerase repeated amplification protocol (Q-TRAP) was used to detect telomerase activity. Cells (1 × 10^6^) were harvested and lysed in NP-40 lysis buffer (1 mM DTT, protease inhibitors, and RNase inhibitor) and diluted to 1 × 10^3^ cells/μL, then centrifuged at 12,000 rpm for 10 min at 4 ℃. The supernatant was collected and the protein concentration of each sample was measured. Typically, 1–3 μg of the protein lysate was detected by PCR. Each 20 μL Q-TRAP reaction contained 2 μL of the eluted proteins, primers, 1 mM EGTA, and GoTaq^®^ qPCR Master Mix (Promega). The reaction mixtures were incubated at 30 ℃ for 30 min and then PCR-amplified using an ABI QuantiStudio 7 Flex real-time PCR system (USA). The amplification program is 90 s at 94 ℃; 40 cycles of 30 s at 94 ℃ and 60 ℃ each [28].

TS primer: 5′-AATCCGTCGAGCAGAGTT-3′.

ACX primer: 5′-GCGCGG(CTTACC)3CTAACC-3′.

### Transmission electron microscopy (TEM)

Cells were suspended in the fixation solution overnight at 4 ℃ and processed by the electron microscopy service center at core facilities for medical science of SYSU following a conventional protocol. The samples were observed using electron microscopy (JEM-1400, JEOL, Japan) at 15,000× magnification.

### In vivo subcutaneous tumor growth assay

BALB/c nude mice were subcutaneously injected with 1 × 10^6^ stable MCCC2 KD HCT116 cells and control cells; each group contained six mice. After 11 days of monitoring, the mice were sacrificed and the tumors were harvested. All the specimens were fixed in formalin and embedded in paraffin for slide preparation. The slides were stained with H&E, anti-Ki67, and anti-MCCC2 antibodies.

### Statistical analysis

Statistical analyses were performed using GraphPad Prism 9.3.0 (RRID:SCR_002798) (Chicago, IL., USA) or SPSS 25.0 (RRID:SCR_002865) (California, USA). Associations between MCCC2 expression and clinical variables were analyzed using chi-squared test. Overall survival was analyzed using Kaplan–Meier analysis, and the *p* value was calculated using the log-rank test. A Cox regression analysis model was used to evaluate univariate and multivariate survival analyses. Differences between groups were analyzed using two-tailed Student’s *t*-test. Two categorical independent variables in multiple comparisons were analyzed by two-way ANOVA. Significance was set at *p* < 0.05.

## Results

### MCCC2 promoted cell proliferation, invasion, and migration in vitro

**Fig. 1:**
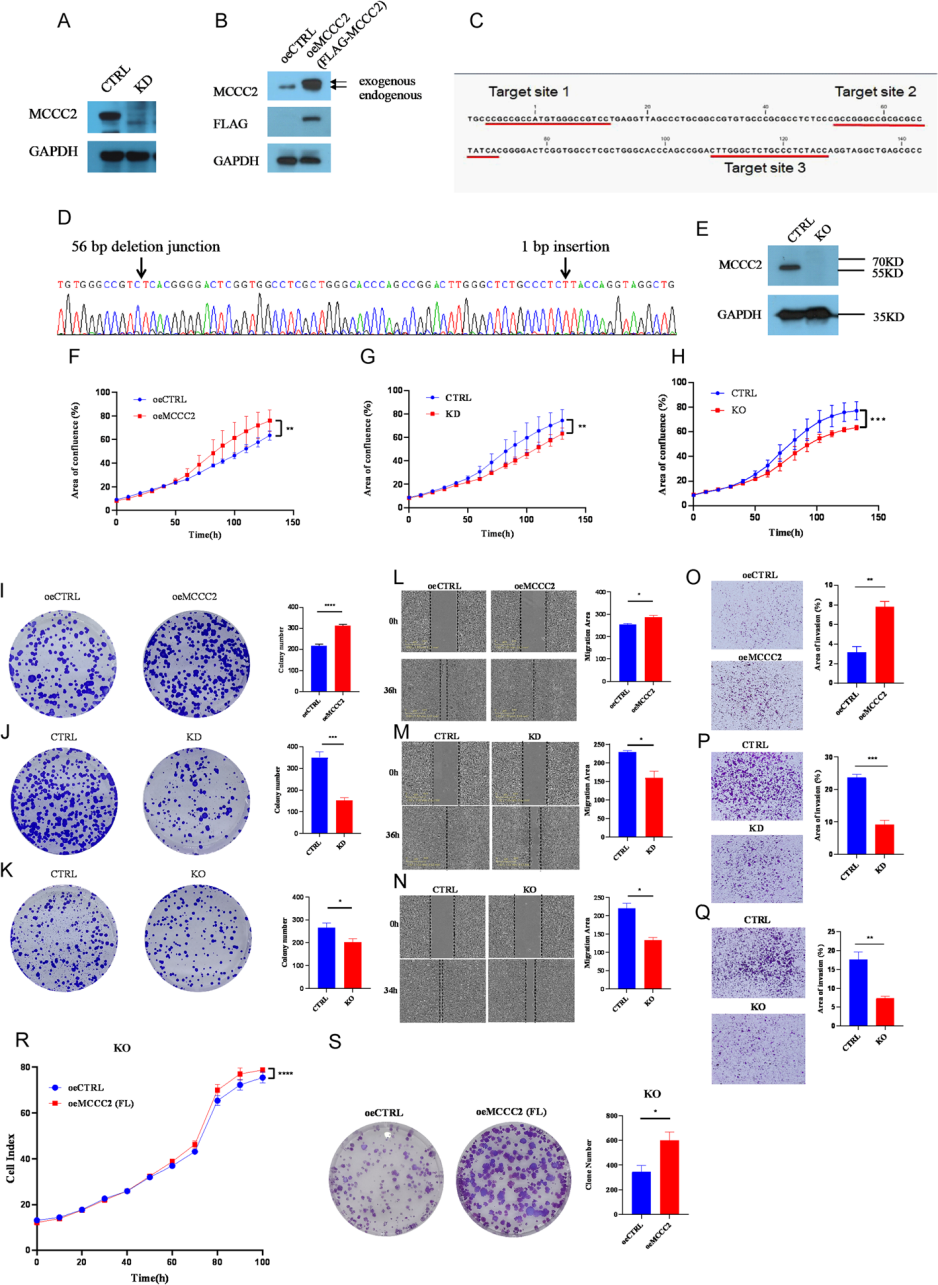
MCCC2 promoted cell proliferation, invasion, and migration in vitro. **A** Western blotting confirmed the knockdown efficiency in HCT116 stable cell line transfected with MCCC2 shRNA or control plasmid. **B** Western blotting confirmed the overexpression of MCCC2 in HCT116 stable cell line transfected with FLAG-MCCC2 or control plasmid. Arrows indicated the exogenous and endogenous form of MCCC2 protein, respectively. **C** Three sgRNAs targeting sites were locating in exon 1 of MCCC2 gene (ATG being the initiation codon). **D** After transfection, and selection, one particular single clone was identified by Sanger sequencing to harbor homozygous 56 bp deletion plus 1 bp insertion in exon 1 of MCCC2 gene, resulting in premature termination codon. **E** Western blotting to confirm the knock out (KO) of MCCC2 gene in **D**). **F**–**H** Proliferation assay by RTCA in pairwise cell lines showed that MCCC2 selective expression could affect cell proliferation. Two‐way ANOVA was used to calculate *p* value. **I**–**K** Representative images and quantification of colony formation assay in pairwise cell lines showed that MCCC2 selective expression could affect colony formation ability (*n* = 3). Paired two-tailed Student’s *t*-test was used to calculate *p* value. **L**–**N** Representative images and quantification of wound healing in pairwise cell lines showed MCCC2 selective expression could affect the cellular migration ability (*n* = 3). Paired two-tailed Student’s *t*-test was used to calculate *p* value. **O**–**Q** Representative images and quantification of Transwell assay in pairwise cell lines showed that MCCC2 selective expression could affect the cellular invasion ability (*n* = 3). Paired two-tailed Student’s *t*-test was used to calculate *p* value. **R** Proliferation assay by IncuCyte showed that after re-expression of MCCC2 in the KO cells enhanced cell proliferation. Two‐way ANOVA was used to calculate *p* value. **S** Representative images and quantification of colony formation assay showed that re-expression of MCCC2 in the KO cells enhanced colony formation ability (*n* = 3). Paired two-tailed Student’s *t*-test was used to calculate *p* value. (All data represent the mean ± SEM. *p < 0.05, **p < 0.01, ***p < 0.001, ****p < 0.0001)

To explore whether MCCC2 expression effects cell proliferation, invasion, and migration, we firstly checked MCCC2 expression level across the Expression Atlas in EMBL-EBI website and estimated the MCCC2 mRNA expression level in eight CRC cell lines (Additional file 1: Fig. S1A, B) the HCT116 cell line with medium MCCC2 level was chosen to construct MCCC2-expressing cell lines, i.e., stably expressing short hairpin RNAs (shRNAs, knockdown: KD, Fig. 1A) and FLAG-MCCC2 overexpressing plasmid (overexpressing: oeMCCC2, Fig. 1B). MCCC2 knockout (KO) using the CRISPR/Cas9 system was also generated in the HCT116 cell line by designing three guide RNA sites targeting the first exon of the MCCC2 gene (Fig. 1C). Successful KO clones were confirmed using Sanger sequencing (Fig. 1D) and western blotting (Fig. 1E). Compared with control cells, overexpression of MCCC2 promoted cell proliferation (Fig. 1F), colony formation (Fig. 1I), cell migration (Fig. 1L), and invasion ability (Fig. 1O), whereas MCCC2 deficiency by either KD or KO inhibited cell proliferation (Fig. 1G, H), colony formation (Fig. 1J, K), cell migration (Fig. 1M, N), and invasion ability (Fig. 1P, Q). Rescue experiments by reexpressing MCCC2 in a KO background restored cell proliferation (Fig. 1R) and colony formation potential (Fig. 1S). The above-mentioned proliferation, migration, and invasion assays were performed in another cell line DLD1 with consistent results (Additional file 1: Fig. S1C–F). Together, these data suggest that MCCC2 exerts oncogenic activity in vitro.

### MCCC2 deficiency induces mitochondrial fusion

**Fig. 2:**
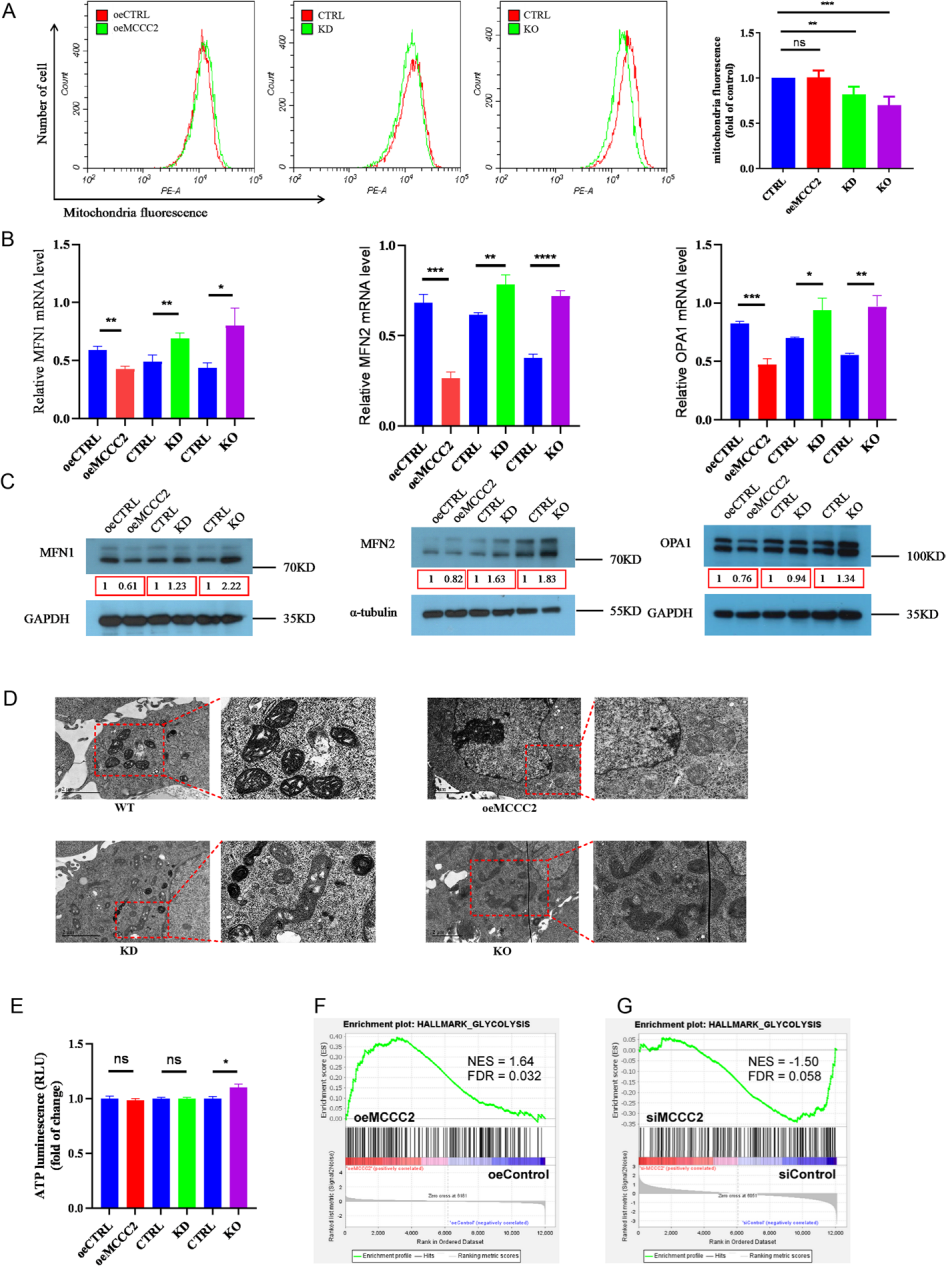
MCCC2 deficiency induced mitochondrial fusion in CRC cells. **A** oeMCCC2, KD, and KO cells with respective control staining by the MitoTracker probe were determined to reflect mitochondrial quantity by flow cytometry. MitoTracker probe staining revealed that MCCC2 deficiency led to a decreased number of mitochondria. **B** Relative mRNA levels of MFN1, MFN2, and OPA1 were quantified using RT-qPCR. MCCC2 deficiency led to the upregulation of mitochondrial fusion markers, whereas MCCC2 overexpression downregulated the expression of these markers. **C** Western blotting confirmed the protein levels of MFN1, MFN2, and OPA1 in oeMCCC2, KD, and KO cells and their controls, respectively. α-Tubulin and GAPDH served as loading controls. Numbers in the box indicate the relative levels in pairwise semiquantification. **D** The mitochondria of oeMCCC2, KD, or KO cells, and control cells, were observed by TEM. The images of mitochondria were taken at 15,000× magnification, and a portion of them was enlarged on the right. MCCC2 deficiency promotes mitochondrial fusion, whileMCCC2 overexpression has no apparent effect on mitochondrial morphology. Scale bar: 2 μm. **E** ATP contents of oeMCCC2, KD, KO cells, and their controls were measured using a luminometric assay and compared in a pairwise fashion. **F** The GSEA analysis of the transcriptomes (oeMCCC2 versus oeControl cells) showed a significant association between oeMCCC2 and hallmark of glycolysis signaling pathway. NES, normalized enrichment score; FDR, false discovery rate. **G** The GSEA analysis of the transcriptomes (siMCCC2 versus siControl cells) showed a significant association between siControl and the hallmark of the glycolysis signaling pathway. All data presented as mean ± SEM (*n* = 3). *p < 0.05, **p < 0.01, ***p < 0.001, ****p < 0.0001

As MCCC2 is known to localize predominately in mitochondria, we investigated whether MCCC2 deficiency would change mitochondrial quantity, morphology, and membrane potential, which are critically related to mitochondrial functions. Mitochondrial numbers were estimated using mitochondria-specific fluorescence probe (MitoTracker red) and flow cytometry. Compared with control cells, total mitochondrial fluorescence intensities were significantly decreased in both MCCC2 KD and KO cells, but not in MCCC2 overexpressing cells (Fig. 2A). We then asked whether mitochondrial morphology, especially the fusion and fission state, changed with changes in MCCC2 levels. Mitofusin 1 (MFN1), mitofusin 2 (MFN2), and OPA1 are critical regulators and markers of mitochondrial fusion. The mRNA levels of MFN1/MFN2/OPA1 were significantly downregulated in MCCC2 overexpressing cells, and were significantly upregulated in MCCC2 KD/KO cells (Fig. 2B). Similar changes in protein levels were observed using western blotting (Fig. 2C). Transmission electron microscopy (TEM) was used to investigate the mitochondrial morphology. While control cells and oeMCCC2 cells showed uniform size and individual round-shaped mitochondria, mitochondrial fusion and swelling were commonly seen in both MCCC2 KD and MCCC2 KO cells (Fig. 2D). Interestingly, although the mitochondria were visualized more in the fusion state, the collective gross ATP in these different cells was comparable at a similar level, with MCCC2 KO cells even higher than control cells (Fig. 2E). MCCC2 KD, oeMCCC2, and control cells were subjected to transcriptome analysis. GSEA enrichment analysis revealed that glycolysis-related genes were positively correlated with MCCC2 expression in both oeMCCC2 versus oeControl comparison (Fig. 2F) and siMCCC2 versus siControl comparison (Fig. 2G). Collectively, these findings suggest that MCCC2 KD or KO resulted in the upregulation of mitochondrial fusion markers and shifted the balance of mitochondrial dynamics towards a fusion state, while maintaining gross ATP production, and MCCC2 may render the cells prefer glycolysis for energy production.

### MCCC2 deficiency leads to telomere shortening in CRC cells

**Fig. 3:**
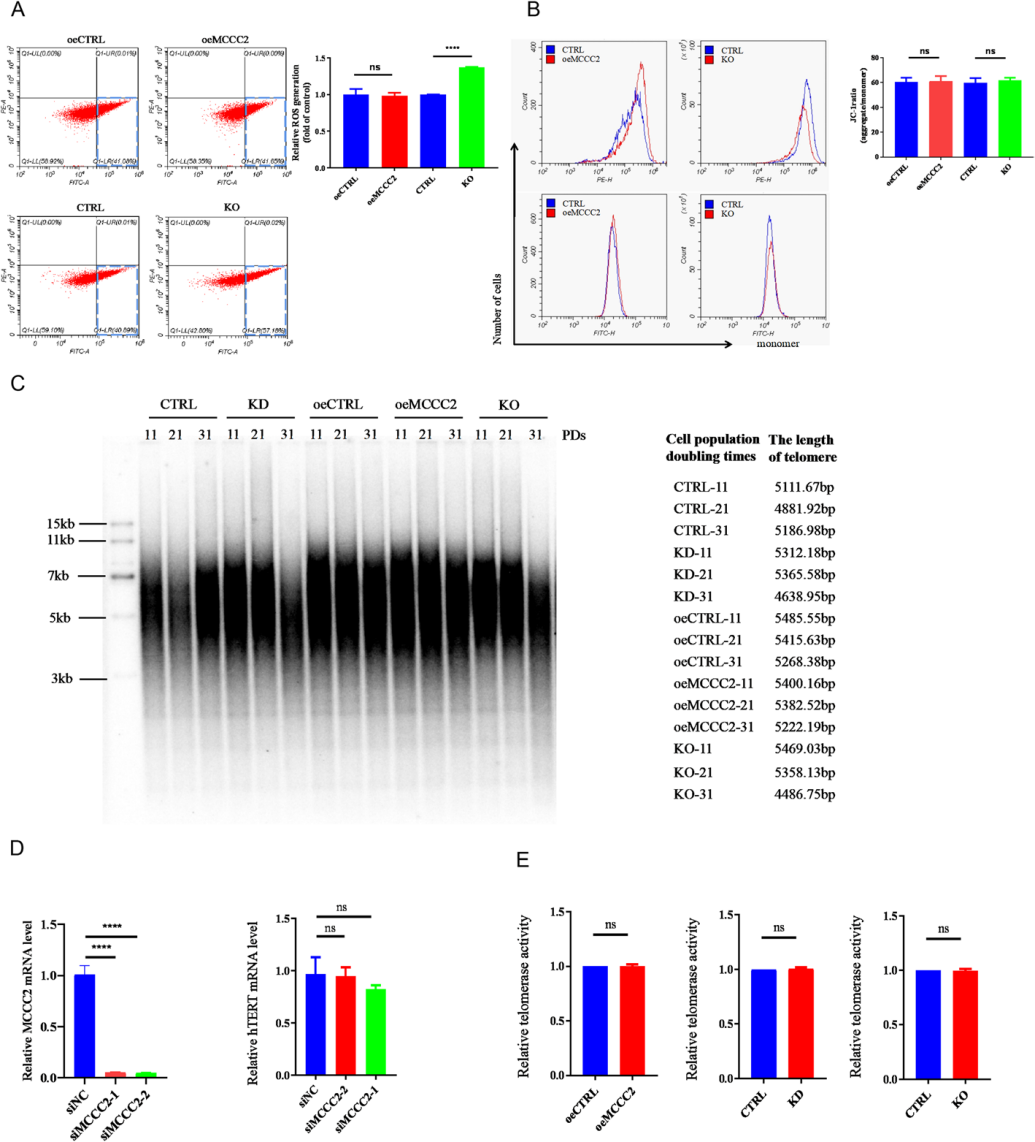
MCCC2 deficiency led to telomere shortening. **A** Reactive oxygen species (ROS) amounts were detected by flow cytometry in oeMCCC2, KO cells, and their controls respectively. **B** Mitochondrial membrane potential in different cells was evaluated by flow cytometry with JC-1 staining method. **C** oeMCCC2, KD, KO cells, and their controls were cultured for the indicated population doublings (PDs) and then subjected to the telomere restriction fragment (TRF) analysis. The length of telomere were calculated based on the phosphor imaging by TeloRun software. **D** Relative mRNA level of hTERT was quantified by RT-qPCR. **E** The relative telomerase activity in oeMCCC2, KD, KO cells, and their controls were quantified by Q-TRAP assay. Paired two-tailed Student’s *t*-test was used to calculate *p* value. All data presented as mean ± SEM (*n* = 3). *p < 0.05, **p < 0.01, ***p < 0.001, ****p < 0.0001

As MCCC2 KD or KO induces mitochondrial fusion, we wondered whether this would cause other mitochondrial dysfunction and lead to ROS generation, which is linked to genomic instability resulting from oxidative DNA damage [29, 30]. Therefore, the ROS levels in different cells were quantified and compared. While oeMCCC2 cells had equal levels of ROS as control cells, MCCC2 KO cells had a significantly higher level of ROS (Fig. 3A). Although more ROS were generated in MCCC2 KO cells, the mitochondrial membrane potential (Δ*ψ*_m_), as revealed by the JC-1 assay, was still comparable to that in control cells (Fig. 3B), suggesting that apoptosis caused by aberrant mitochondrial membrane potential was unlikely to be triggered in MCCC2 KO cells. To examine whether MCCC2 contributes to telomere maintenance, telomere restriction fragment (TRF) assays were performed to compare telomere lengths in different cell lines. The results showed that after 31 rounds of population doubling (PDs), the telomere lengths of MCCC2 KD and KO cells were shorter than those of control cells (Fig. 3C). The rate of telomere loss was converted to approximately 20–30 bp per population doubling in the KO cells. MCCC2 KD by siRNA did not cause a significant change in TERT transcriptional level (Fig. 3D). We also measured the total telomerase activity by Q-TRAP, and the results showed that both oeMCCC2 and KD/KO cells had similar levels of telomerase activity compared with control cells (Fig. 3E). These results indicated that MCCC2 deficiency caused more ROS generation, and was linked to shorter telomeres, without affecting TERT level and telomerase activity.

### MCCC2 forms a complex with TRF2

**Fig. 4:**
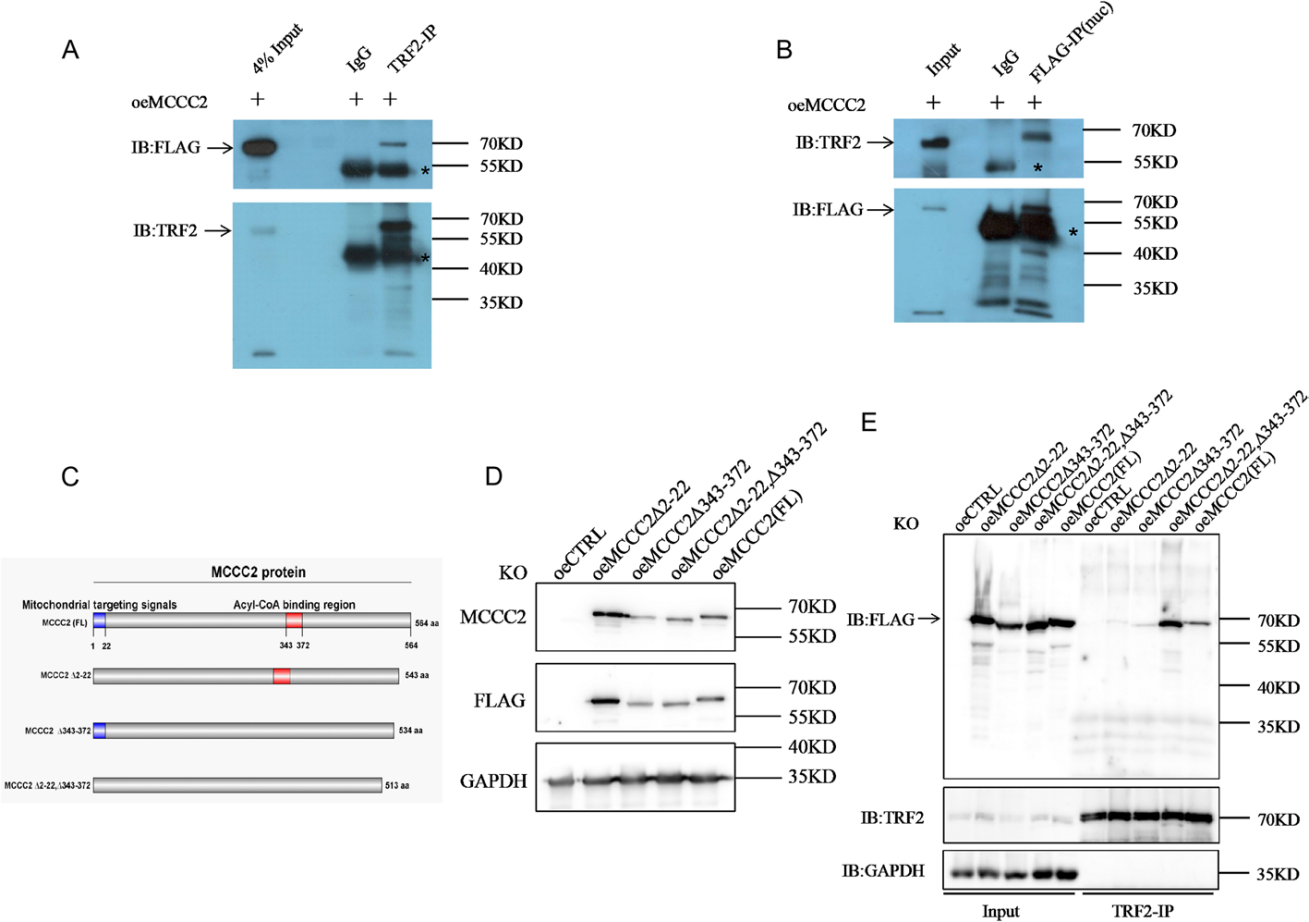
MCCC2 formed a complex with the shelter in protein. **A** Co-immunoprecipitation (Co-IP) experiment was performed by applying the anti-TRF2 antibody from MCCC2-FLAG expressing cell extract, and IgG served as a negative control. The result showed that endogenous TRF2 formed a complex with exogenous MCCC2. Star indicates unspecific band nearby the target band. **B** Co-IP experiment was performed by applying the anti-FLAG antibody from MCCC2-FLAG expressing nucleus extract with the nucleus and cytoplasm separated kit. The result showed that exogenous MCCC2 interacted with endogenous TRF2. Star indicates unspecific band nearby the target band. **C** Scheme of the human MCCC2 protein motifs: 2–22, Mitochondrial targeting signals; 343–372, Acyl-CoA binding region. ∆2–22 and ∆343–372 indicate two individual construct with the corresponding motif deleted. **D** MCCC2 KO cells were stably transfected with FLAG-Tagged full-length and FLAG-Tagged mutants (∆2–22 or/and ∆343–372) of MCCC2 plasmids. Western blotting analysis suggested that they were all able to express at a similar level in the cells. **E** Cell lysates from (**D**) were subjected to Co-IP experiment by applying the anti-TRF2 antibody. The result showed that endogenous TRF2 formed a complex with mutant MCCC2

MCCC2 KD or KO did not affect TERT levels and activity, a crucial and major mechanism for telomere maintenance. Telomeres are protected in part by a telomere-associated protein complex, that is, the shelterin complex, in which TRF2 is a DNA direct binding factor. A previous study using high-throughput LC–MS/MS analysis reported that MCCC2 may form a complex with TRF2 [31]. We next confirmed whether MCCC2 forms a complex with TRF2 using co-immunoprecipitation (CO-IP) assays. Endogenous TRF2 co-immunoprecipitated with MCCC2 (Fig. 4A). Exogenously expressed MCCC2 (FLAG-tagged) also co-immunoprecipitated with endogenous TRF2 (Fig. 4B). MCCC2 contains a mitochondrial targeting signal and an acyl-CoA binding region (illustrated in Fig. 4C). To confirm whether these regions are critical for TRF2 binding, FLAG-tagged mutants of MCCC2 were expressed in MCCC2 KO cells (Fig. 4D) and subjected to CO-IP analysis as described above. Endogenous TRF2 was still able to co-immunoprecipitate with the mutant MCCC2 protein (Fig. 4E), suggesting that these two regions are dispensable for MCCC2-TRF2 complex formation in cells.

### MCCC2 was upregulated in CRC and associated with longer OS

**Fig. 5:**
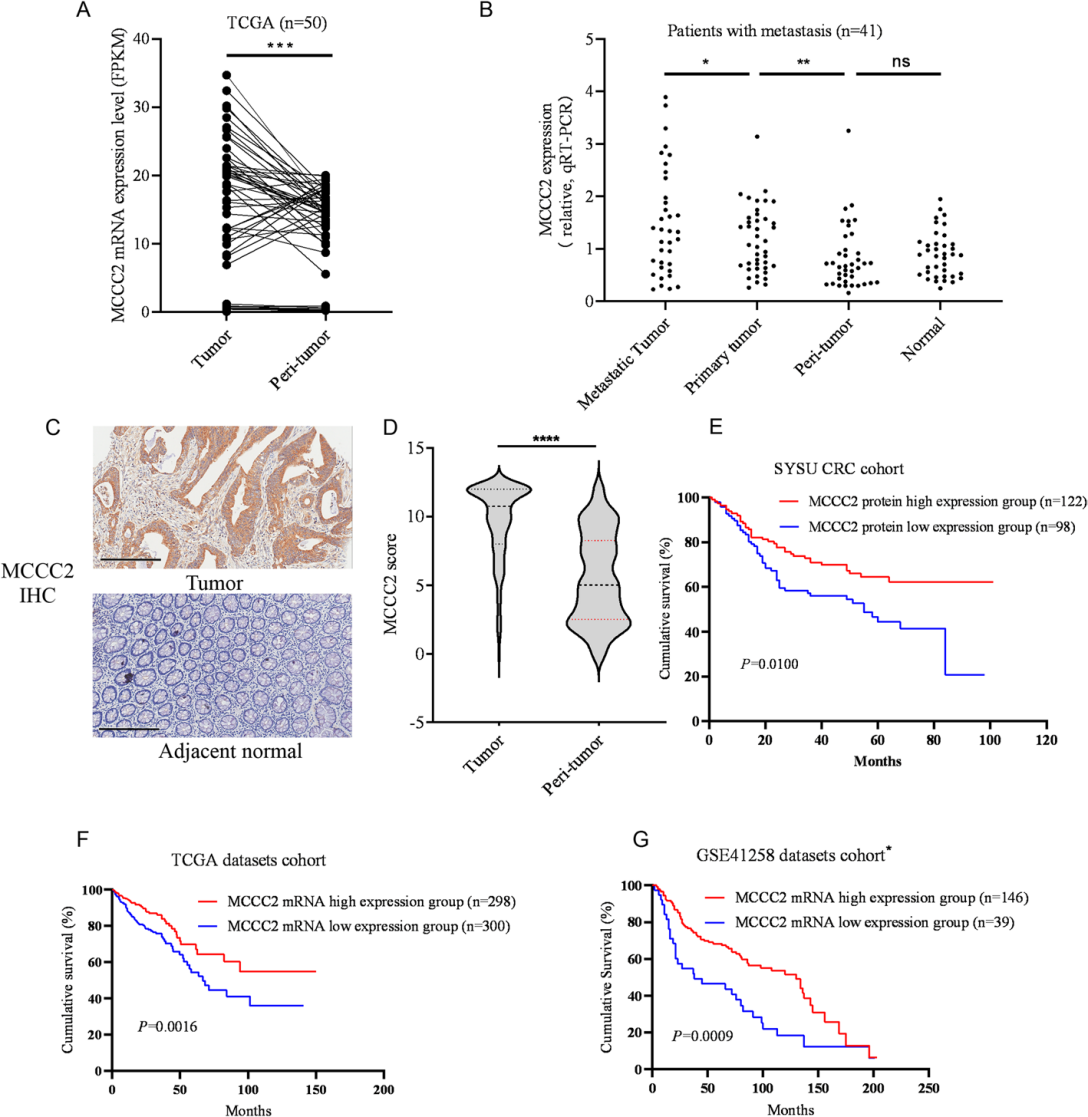
MCCC2 was upregulated in CRC and associated with longer OS. **A** The mRNA level of MCCC2 was significant higher in CRC tissues than in peritumor tissues in the TCGA cohorts (*n* = 50). Paired two-tailed Student’s *t*-test was used to calculate *p* value, ***p < 0.001. **B** The mRNA levels of MCCC2 in primary tumors, matched peritumor, metastatic tumor, and normal tissues of CRC patients (*n* = 41) were quantified by RT-qPCR and compared. Paired two-tailed Student’s *t*-test was used to calculate *p* value,*p < 0.05, **p < 0.01. **C** Representative image of IHC staining of MCCC2 in CRC tissue and adjacent tissue were showed. Scale bars: 200 µm. **D** IHC scores for MCCC2 in a tissue microarray of CRC patients (*n* = 210) were analyzed. Paired two-tailed Student’s *t*-test was used to calculate *p* value, ****p < 0.0001. **E** Kaplan–Meier survival curves of overall survival based on IHC scores of MCCC2 in the tissue microarray. The MCCC2 protein high expression group was associated with longer OS than the MCCC2 protein low expression group (*p* = 0.01). **F** Survival curves based on TCGA database showed that patients with high MCCC2 mRNA expression survived significantly longer than those with low MCCC2 mRNA expression (*p* = 0.0016). **G** Survival curves based on GEO41258 datasets showed that patients with high MCCC2 mRNA expression survived significantly longer than those with low MCCC2 mRNA expression (*p* = 0.0009). *Data collection only included patients with carcinoma in situ

Next, we investigated the significance of MCCC2 expression in CRC cells. First, by analyzing TCGA data (50 CRC cases with tumor and peritumor tissues self-matched), we found that the mRNA level of MCCC2 was significantly higher in tumor tissues than in peritumor tissues (Fig. 5A). We then detected the mRNA level of MCCC2 in metastatic tumor tissues, primary tumor tissues, peritumor tissues (~ 2 cm from the tumor site), and normal tissues (~ 5 cm from the tumor site) in 41 CRC patients’ samples independently. The results showed that MCCC2 expression was higher in CRC tumor tissues than in peritumoral and normal tissues. Moreover, the mRNA level of MCCC2 was the highest in metastatic tumor tissues (Fig. 5B). To further investigate the association between MCCC2 expression and the clinical characteristics of the patients, we examined the tissue microarray of 233 CRC patients using IHC for MCCC2 protein levels. High MCCC2 protein expression was positively associated with sex (*p* = 0.028), depth of invasion (*p* = 0.008), distant metastasis (*p* < 0.001), TNM stage (*p* = 0.008), and vein invasion (*p* < 0.001) in CRC patients. However, there was no significant correlation between MCCC2 and age, location, histological grade, lymph node metastasis, nerve invasion, and tumor size (*p* > 0.05, Table 1). The staining of positive expression of MCCC2 was mainly concentrated in the cytoplasm of CRC tissues. In contrast, the adjacent normal glands presented negative or low levels (Fig. 5C). The IHC scores were similar to the MCCC2 mRNA levels, and the IHC scores in tumor tissues were significantly higher than those in the adjacent normal tissues (Fig. 5D). The patient group with higher MCCC2 expression had longer overall survival time than the group with relatively low MCCC2 expression (Fig. 5E), similar to the analysis results from the TCGA CRC cohort (Fig. 5F) and GSE41258 CRC cohort (Fig. 5G) (https://portal.gdc.cancer.gov/ and https://www.ncbi.nlm.nih.gov/geo/). Cox regression analyses revealed that age (*p* = 0.001), distant metastasis (*p* < 0.001), and histological grade (*p* = 0.008) were independent prognostic factors for patients with CRC. However, MCCC2 expression was not an independent predictor of OS in CRC patients (Table 2).

**Table 1:** Correlation between MCCC2 expression and clinicopathological features of CRC patients

Characteristic	MCCC2		
	Low expression (*n* = 98)	High expression (*n* = 112)	*p*-Value
Age	1		0.581
< 60	54 (55.1)	57 (50.89)	
≥ 60	44 (44.9)	55 (49.12)	
Gender			0.018
Female	34 (34.7)	58 (51.8)	
Male	64 (65.3)	54 (48.2)	
Location			1.000
Colon	80 (81.6)	91 (81.2)	
Rectum	18 (18.4)	21 (18.8)	
Histological grade			0.028
Well	23 (23.5)	19 (17.0)	
Moderately	49 (50.0)	76 (67.9)	
Poorly	26 (26.5)	17 (15.2)	
Depth of invasion			0.008
T1	6 (6.1)	5 (4.5)	
T2	10 (10.2)	14 (12.5)	
T3	41 (41.8)	24 (21.4)	
T4	41 (41.8)	69 (61.6)	
Lymph node metastasis			0.249
N0	28 (28.6)	41 (36.6)	
N1	46 (46.9)	53 (47.3)	
N2	24 (24.5)	18 (16.1)	
Distant metastasis			< 0.001
M0	30 (30.6)	65 (58.0)	
M1	68 (69.4)	47 (42.0)	
TNM stage			0.008
I	12 (12.2)	12 (10.7)	
II	9 (9.2)	21 (18.8)	
III	9 (9.2)	32 (28.6)	
IV	68 (69.4)	47 (42.0)	
Vein invasion			< 0.001
Yes	20 (20.4)	21 (18.8)	
No	78 (79.6)	91 (81.2)	
Nerve invasion			0.534
Yes	29 (29.6)	28 (25.0)	
No	69 (70.4)	84 (75.0)	
Tumor size			0.441
≤ 5 cm	74 (75.5)	79 (70.5)	
> 5 cm	24 (24.5)	33 (29.5)

**Table 2:** Univariate and multivariate analyses of different prognostic parameters of the CRC patients in the cohort

Factor	Univariate analysis	*p*-Value	Multivariate analysis	*p*-Value
	HR (95% CI)		HR (95% CI)	
Age (≤ 60/ > 60)	0.615 (0.395–0.957)	0.031	2.203 (1.387–3.497)	0.001
Gender (female/male)	0.854 (0.546–1.335)	0.488		
Location (colon/rectum)	0.682 (0.374–1.245)	0.212		
T stage (T1 + T2/T3 + T4)	0.176 (0.069–0.446)	< 0.001		
N stage (N0/N1 + N2)	0.293 (0.163–0.524)	< 0.001		
Distant metastasis (absent/present)	0.083 (0.042–0.167)	< 0.001	12.138 (6.024–24.458)	< 0.001
TNM stage (I + II/ III + IV)	0.171 (0.078–0.375)	< 0.001		
Histological grade (well + moderate/poor)	0.450 (0.277–0.733)	0.001	1.995 (1.196–3.327)	0.008
Vein invasion (no/yes)	0.647 (0.386–1.085)	0.099		
Nerve invasion (no/yes)	0.503 (0.317–0.796)	0.003		
Tumor size (≤ 5/ > 5 cm)	0.663 (0.416–1.055)	0.083		
MCCC2 expression (low/high)	1.661 (1.067–2.586)	0.025		

### MCCC2 KD inhibited tumor growth in vivo

**Fig. 6:**
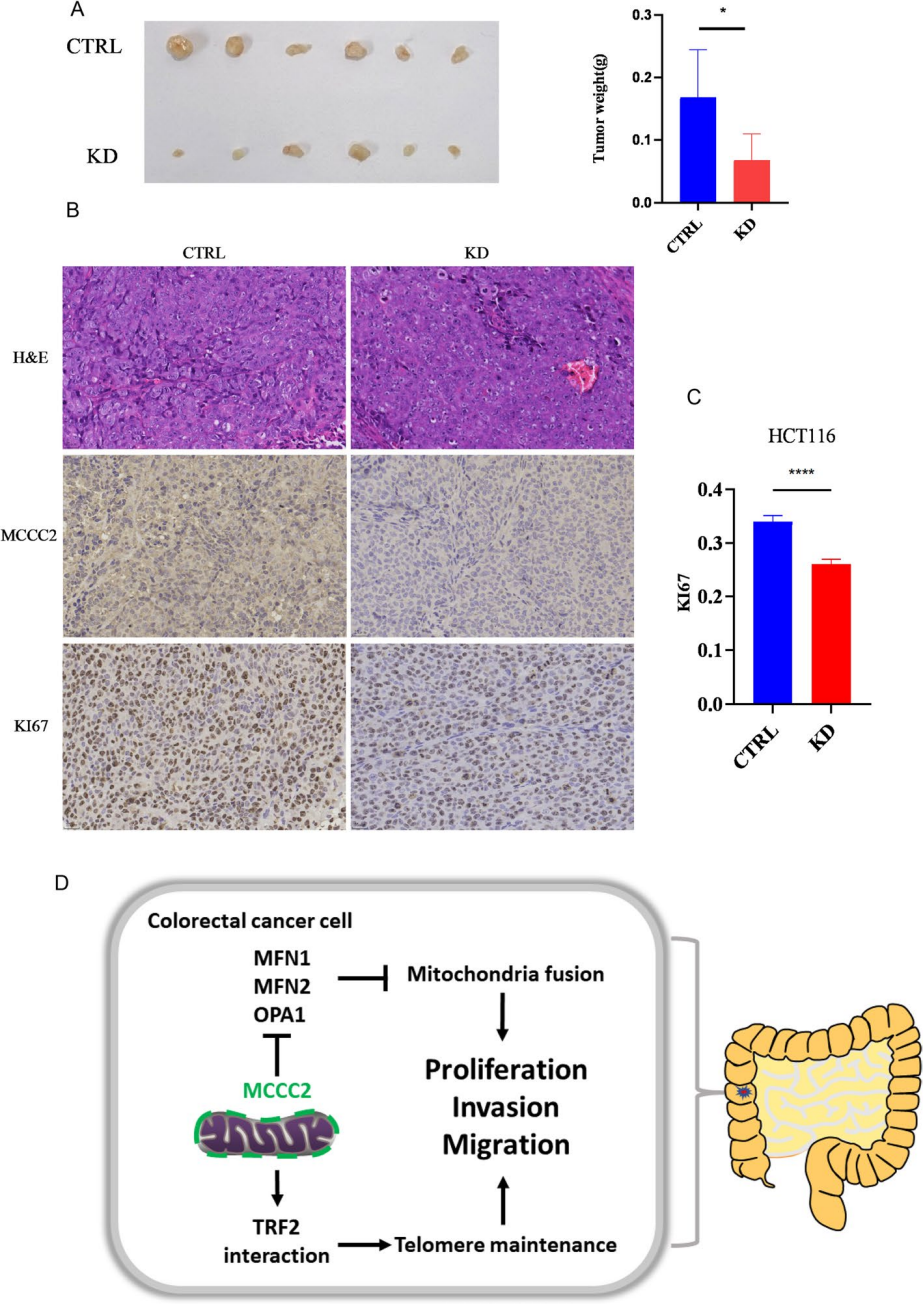
MCCC2 KD inhibited CRC cell proliferation in vivo. **A** 1 × 10^6^ of HCT116 cells with MCCC2 stably KD and its control cells were injected into nude mice subcutaneously (*n* = 6 per group). Tumors were harvested on day 11. The weights of tumors formed from KD group were lower than the control group. Paired two-tailed Student’s *t*-test was used to calculate *p* value. Data represent the mean ± SEM (*n* = 6). *p < 0.05. **B** Sectioned tumor samples from animals were subjected to H&E staining, IHC staining with anti-MCCC2 and anti-Ki-67 antibody, and representative images are showed. **C** Quantification of Ki-67 staining in (**C**). Paired two-tailed Student’s *t*-test was used to calculate *p* value. Data represent the mean ± SEM (*n* = 3). ****p < 0.0001. **D** Integrative diagram for MCCC2 in mitochondria regulation, telomere maintenance, and CRC cellular behaviors. MCCC2 inhibited the expression of MFN1/MFN2/OPA1, which in turn inhibited mitochondria fission that is beneficial for cellular energy production; MCCC2 could also form a complex with TRF2, playing potential role in telomere maintenance. Together, MCCC2 expression promoted CRC proliferation, invasion, and migration

To confirm the effect of MCCC2 deficiency on tumor growth in vivo, a tumor formation model was established in nude mice by subcutaneous injection of cancer cells. When the tumors were visible after 11 days, the mice were sacrificed and tumor weight was measured. Tumor weight was significantly reduced in the MCCC2 KD mice group (Fig. 6A). The tumor samples were sectioned and subjected to IHC staining for MCCC2 and Ki-67. The MCCC2 level was indeed much lower in the KD group, and tumor cell proliferation rate was also lower in the KD group revealed by Ki-67 staining and counting (Fig. 6B, C). Taken together, the importance of MCCC2 repression in mitochondrial regulation, telomere maintenance, and CRC cellular behaviors is summarized and illustrated (Fig. 6D).

## Discussion

Our data demonstrated that both MCCC2 KD and KO enhanced mitochondrial fusion in CRC cells. It has been showed that oncogenic cancer cells lead to diminished oxidative phosphorylation (OXPHOS) but, rather, rely on aerobic glycolysis to generate energy, which is termed the “Warburg effect” [9, 32]. In human skin fibroblast cells, it was demonstrated that MCCC1 deficiency would repress OXPHOS, decrease ATP production, and increase glycolysis capacity, a phenomenon similar to that in CRC cells [33]. Oncogenic signaling can facilitate mitochondrial fission to adapt to the increasing demand for aerobic glycolysis. Meanwhile, mitochondrial fusion causes an intermixing of matrix contents, including mitochondrial proteins, mtDNA, and nutrients, which maintains mitochondrial homogeneity and OXPHOS by diluting the dysfunctional proteins and aberrant mtDNA. MCCC2 protein is mainly located in mitochondria, and MCCC2, KD, and KO may engender dysfunction proteins and aberrant mtDNA to promote mitochondrial fusion. Regrettably, it may be challenging to detect if mitochondrial fission occurs in MCCC2 overexpression CRC cells, given that mitochondria are widely distributed in cancer cells.

The altered extranuclear activity of the catalytic subunit of telomerase TERT in non-canonical functions can play a protective role against ROS injury and decrease apoptosis. TERT can bind to mitochondrial DNA (mtDNA) to protect it from oxidative damage [34]. Moreover, the hTERT promoter region contains many cis-elements involved in transcriptional regulation, such as c-MYC, SP1, p53, etc. Many TERT transcriptional regulators are actively involved in cell proliferation, differentiation, self-renewal, and apoptosis [35]. Although the primary function of TERT is telomere re-lengthening, TERT copy number amplification associated with telomerase activity occurs in multiple malignancies [36]. In our study, MCCC2 deficiency did not affect telomerase transcription or activity in CRC cells.

Although we showed that MCCC2 and TRF2 could form a complex in the cell, where, when, and how they interact remains unknown. Telomeric-repeat-containing RNA(TERRA) is transcribed from chromosomal ends and regulates telomeric chromatin structure and telomere maintenance through telomerase. It interacts with several telomeric proteins such as TRF1 and TRF2 [37]. MCCC2 has also been showed to interact with TERRA using SILAC-based RNA–protein interaction analysis [38]. It is tempting to speculate that a complex of MCCC2–TRF2–TERRA exists, with TERRA as the scaffold, and this complex might be critical for telomerase recruitment to telomeres. If this particular scenario is plausible, MCCC2 deficiency may lead to insufficient telomerase recruitment, resulting in telomere attrition. All three MCCC2 functional domain mutants were able to form complexes with full-length TRF2. Pinpointing the specific domains responsible for the MCCC2-TRF2 interaction should shed light on molecular basics. The next step is to search for important domains or motifs in TRF2 that interacts with MCCC2.

We also found that MCCC2 played an important role in CRC. MCCC2 expression was significantly higher in CRC tumor tissues compared with in adjacent normal tissues, which was consistent with the TCGA cohort. In metastatic tissues, MCCC2 was upregulated compared with that in tissues without metastasis, according to IHC staining scores. High expression of MCCC2 was associated with longer OS, consistent with results from TCGA cohort analysis. The occurrence and development of tumors involves complex molecular regulation, with gene activation and inactivation being dynamically modulated. The prognostic value of the expression of a specific gene, regardless of whether it is an oncogene or tumor suppressor in general, should be referred to tumor microenvironment. Research has shown that the chemokine ligand C-X-C motif chemokine ligand 11 (CXCL11), also an oncogene, correlates with an improved prognosis in colon cancer [39]. It induces the expression of some cytotoxic genes (IFNG, GZMA, GZMB, GZMK, GZMM, and PRF1) and immunosuppressive molecules, such as PD-L1, to promote antitumor immunity. However, this situation does not occur in patients with rectal cancer [39]. Another oncogene, polo-like kinase (Plk1), is overexpressed in a wide spectrum of cancers, and correlates with improved survival in specific breast cancer subtypes by causing abnormal chromosome segregation and cytokinesis, which leads to polyploid cells with reduced proliferative potential to inhibit tumor growth [40]. MCCC2 may act as a passenger gene in the occurrence and development of CRC, and metastasis to specific organs may be regulated by many factors in multiple ways. We speculated that patients with high MCCC2 expression may have a better response to a certain chemotherapy regimen or a certain treatment modality, resulting in a better prognosis. Therefore, the prognostic value of MCCC2 in CRC patients warrants further validation and exploration.

Mitochondrial dysfunction is a hallmark of several diseases [11]. Moreover, mitochondrial dysfunction involves unbalanced mitochondrial dynamics between fusion and fission states [41]. Mitochondrial dynamics can regulate metabolic processes for energy production, the cell cycle, anabolic growth, and survival in cancer cells [42]. Many studies have reported that mitochondrial fission is enhanced in several types of human cancer cells [43]. Mitochondria architecture and network are also dynamically regulated to meet the energy need in lung cancer [44]. In this study, we found an increase in mitochondrial fusion in CRC cells without MCCC2 or with less MCCC2 protein, which provides indirect evidence that MCCC2 reinforces cancer cell proliferation. However, a detailed molecular mechanism through which MCCC2 triggers mitochondrial fusion is required in future study. This is also true for how MCCC2 deficiency leads to telomere dysfunction. Although we did not observe significant ROS change in oeMCCC2 cells, the possibility that mild change existed could not be ruled out. ROS is linked to telomere damage and dysfunction. This may explain why substantial decreasing of telomere length is observed in oeMCCC2-31, but not as apparent as in KO/KD cells. MCCC2 deficiency may induce telomere abnormality through TRF2, or ROS, or both in a synergistic action. Nonetheless, MCCC2 is involved in both mitochondrial dynamic regulation and telomere maintenance, and serves as a novel mediator in between, inspiring us to unveil the detailed mechanisms in the future.

## Conclusions

Overall, we identified MCCC2 as a novel mediator between mitochondria and telomeres, and provided an additional biomarker for CRC stratification.

### Supplementary Information

**Additional file 1. Fig. S1. **MCCC2 promoted cell proliferation, invasion, and migration in vitro. **A** The MCCC2 expression level in human CRC cell line based on Expression Altas database from EMBL-EBL website. **B** The relative mRNA expression level of MCCC2 in human 8 CRC cell lines analyzed by qRT-PCR**. C** Proliferation assay using IncuCyte in both transient MCCC2 overexpression by cell transfection of MCCC2-FLAG plasmids and transient siRNA MCCC2 in DLD1 cells showed that MCCC2 selective expression could affect cell proliferation in DLD1 cells. Two‐way ANOVA was used to calculate *p* value. **D**Representative images and quantification of colony formation assay in both transient MCCC2 overexpression by cell transfection of MCCC2-FLAG plasmids and transient siRNA MCCC2 in DLD1 cells showed that MCCC2 selective expression could affect cell proliferation ability (*n* = 3). A paired two-tailed Student’s *t*-test was used to calculate *p* values.** E** Representative images and quantification of wound healing in both transient MCCC2 overexpression by cell transfection of MCCC2-FLAG plasmids and transient siRNA MCCC2 in DLD1 cells showed that MCCC2 selective expression could affect cell migration ability (*n* = 3). A paired two-tailed Student’s *t*-test was used to calculate *p* value. F Representative images and quantification of Transwell assay in both transient MCCC2 overexpression by cell transfection of MCCC2-FLAG plasmids and transient siRNA MCCC2 in DLD1 cells showed that MCCC2 selective expression could affect the invasion ability (*n* = 3). A paired two-tailed Student’s *t*-test was used to calculate *p* value. (All data are represented as the mean ± SEM. *p < 0.05, **p < 0.01, ***p < 0.001, ****p < 0.0001)

## Data Availability

All data associated with the current study are presented in the manuscript or Supplementary Data. Original materials are available from the corresponding author on rational request.

## Abbreviations

Co-IP: Co-immunoprecipitation
TCGA: The Cancer Genome Atlas
CRC: Colorectal cancer
qRT-PCR: Quantitative reverse transcription polymerase chain reaction
IHC: Immunohistochemistry
OS: Overall survival
KD: Knockdown
KO: Knockout
oe: Overexpression
MFN1: Mitofusin 1
MFN2: Mitofusin 2
OPA1: Optic atrophy 1
DRP1: Dynamin-related protein 1
TRF1: Telomeric repeat-binding factor 1
TRF2: Telomeric repeat-binding factor 2
CXCL11: C-X-C motif chemokine ligand 11
DSB: Double-strand break
TERT: Telomerase reverse transcriptase
TERC: Telomeric RNA component
ROS: Reactive oxygen species
TRF: Telomeric restriction fragment
Q-TRAP: QPCR-based telomerase repeated amplification protocol
TEM: Transmission electron microscopy
GSEA: Gene set enrichment analysis
OXPHOS: Oxidative phosphorylation
mtDNA: Mitochondrial DNA
TERRA: Telomeric repeat-containing RNA
PLK1: Polo-like kinase 1
