# Supplementary figures and images for: MCCC2 is a novel mediator between mitochondria and telomere and functions as an oncogene in colorectal cancer
Source: Cell Mol Biol Lett. 2023 Oct 12;28:80. doi: 10.1186/s11658-023-00487-0 (PMC10571261; doi:10.1186/s11658-023-00487-0)

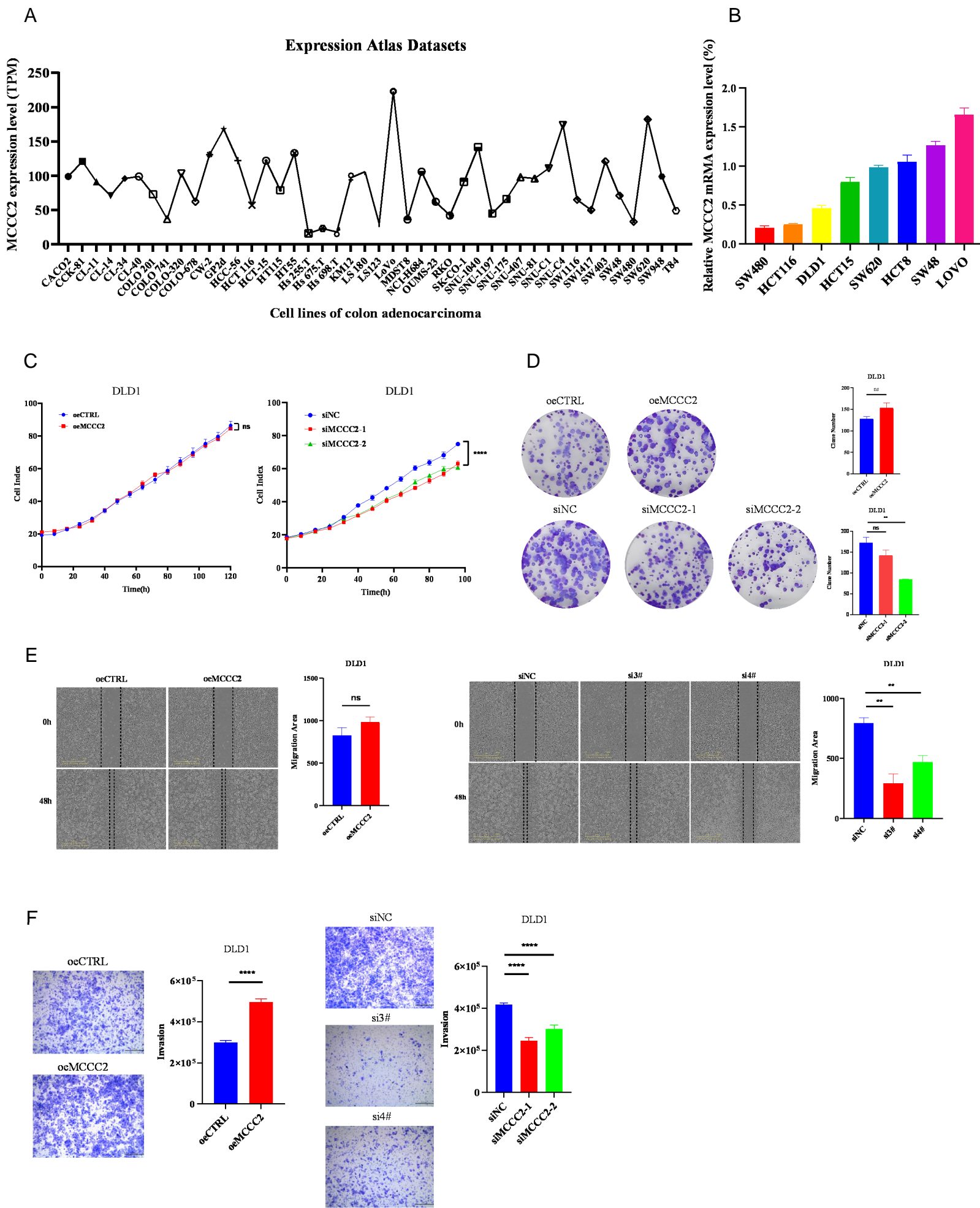

Supplement: Supplementary file 1 — Additional file 1. Fig. S1. MCCC2 promoted cell proliferation, invasion, and migration in vitro. A The MCCC2 expression level in human CRC cell line based on Expression Altas database from EMBL-EBL website. B The relative mRNA expression level of MCCC2 in human 8 CRC cell lines analyzed by qRT-PCR. C Proliferation assay using IncuCyte in both transient MCCC2 overexpression by cell transfection of MCCC2-FLAG plasmids and transient siRNA MCCC2 in DLD1 cells showed that MCCC2 selective expression could affect cell proliferation in DLD1 cells. Two‐way ANOVA was used to calculate p value. DRepresentative images and quantification of colony formation assay in both transient MCCC2 overexpression by cell transfection of MCCC2-FLAG plasmids and transient siRNA MCCC2 in DLD1 cells showed that MCCC2 selective expression could affect cell proliferation ability (n = 3). A paired two-tailed Student’s t-test was used to calculate p values. E Representative images and quantification of wound healing in both transient MCCC2 overexpression by cell transfection of MCCC2-FLAG plasmids and transient siRNA MCCC2 in DLD1 cells showed that MCCC2 selective expression could affect cell migration ability (n = 3). A paired two-tailed Student’s t-test was used to calculate p value. F Representative images and quantification of Transwell assay in both transient MCCC2 overexpression by cell transfection of MCCC2-FLAG plasmids and transient siRNA MCCC2 in DLD1 cells showed that MCCC2 selective expression could affect the invasion ability (n = 3). A paired two-tailed Student’s t-test was used to calculate p value. (All data are represented as the mean ± SEM. *p < 0.05, **p < 0.01, ***p < 0.001, ****p < 0.0001) [file 11658_2023_487_MOESM1_ESM.pdf]
